# THE EFFECT OF FINANCIAL DIFFICULTIES ON COGNITIVE FUNCTION IN A JAPANESE ELDERLY POPULATION SAMPLE

**DOI:** 10.1093/geroni/igz038.1171

**Published:** 2019-11-08

**Authors:** Yumi Ishikawa

**Affiliations:** Osaka School of International Public Policy, Osaka University, Osaka, Japan

## Abstract

Background: Studies have been investigating the effect of financial difficulties on cognitive function especially in the US and Australia. However, there is still a discrepancy regarding the results. This study aims to estimate the effect of financial difficulties on cognitive function in a Japanese elderly population sample. It is rewarding to focus on the Japanese setting, which has the highest proportion of elderly in the world. Method: This study uses a longitudinal panel dataset which include randomly selected elderly Japanese citizens aged 60 and over; the National Survey of the Japanese Elderly. It is a panel dataset containing income and cognitive function. It is ideal dataset to capture the probability of onset of cognitive impairment as it focuses on the elderly. We estimate the effect of participants’ equivalent income on the probability of onset of cognitive impairment at a following survey point using random-effect probit model. Result: The first main result is there is a significant negative effect from financial difficulties on cognitive function. The results indicate that when participants’ equivalent income drops by 1%, they are 2.2% more likely to develop cognitive impairment. The second result is that this negative effect is heterogeneous, depending on their income level. Specifically, this negative effect is observed only at low income level, but not at high income level. That is, the deteriorating effect by impoverishment would be severe when the financially needy people faced income drops. Discussion: Income support plays an important role in improving recipients’ cognitive function, especially among the poor.

